# Thyroid Hormone Regulation of Gene Expression in Primary Cerebrocortical Cells: Role of Thyroid Hormone Receptor Subtypes and Interactions with Retinoic Acid and Glucocorticoids

**DOI:** 10.1371/journal.pone.0091692

**Published:** 2014-03-11

**Authors:** Pilar Gil-Ibáñez, Juan Bernal, Beatriz Morte

**Affiliations:** 1 Instituto de Investigaciones Biomédicas, Consejo Superior de Investigaciones Científicas (CSIC), Madrid, Spain; 2 Universidad Autónoma de Madrid (UAM), Madrid, Spain; 3 Center for Biomedical Research On Rare Diseases (Ciberer), Instituto de Salud Carlos III, Madrid, Spain; Laboratoire de Biologie du Développement de Villefranche-sur-Mer, France

## Abstract

The effects of thyroid hormone on brain development and function are largely mediated by the binding of 3,5,3′-triiodo-L-thyronine (T3) to its nuclear receptors (TR) to regulate positively or negatively gene expression. We have analyzed by quantitative polymerase chain reaction the effect of T3 on primary cultured cells from the embryonic mouse cerebral cortex, on the expression of *Hr*, *Klf9*, *Shh*, *Dio3*, *Aldh1a1*, and *Aldh1a3*. In particular we focused on T3 receptor specificity, and on the crosstalk between T3, retinoic acid and dexamethasone. To check for receptor subtype specificity we used cerebrocortical cells derived from wild type mice and from mice deficient in thyroid hormone receptor subtypes. Receptor subtype specificity was found for *Dio3* and *Aldh1a1*, which were induced by T3 only in cells expressing the T3 receptor alpha 1 subtype. Interactions of T3 with retinoic acid signaling through the control of retinoic acid metabolism are likely to be important during development. T3 had opposing influences on retinoic acid synthesizing enzymes, increasing the expression of *Aldh1a1*, and decreasing *Aldh1a3*, while increasing the retinoic acid degrading enzyme *Cyp26b1*. Dexamethasone increased *Klf9* and *Aldh1a1* expression. The effects of T3 and dexamethasone on *Aldh1a1* were highly synergistic, with mRNA increments of up to 20 fold. The results provide new data on thyroid hormone regulation of gene expression and underscore the importance of thyroid hormone interactions with retinoic acid and glucocorticoids during neural development.

## Introduction

Thyroid hormone action on mammalian brain development is exerted through regulation of gene expression mediated by binding of the active hormone T3 to the nuclear receptors encoded by the *THRA*/*Thra* and *THRB*/*Thrb* genes [1], [2]. The *THRA*/*Thra* gene encodes the T3 binding isoform TRα1, and several non T3-binding splicing products. The *THRB*/*Thrb* gene encodes the T3 biding isoforms TRβ1 and TRβ2. The TR subtypes and isoforms have different but overlapping distribution in tissues, with distinct roles in development and physiology [3].

Hypothyroidism in rodents during the fetal and neonatal period, as well as in adult animals leads to increased or decreased expression of many genes in the cerebral cortex and other brain regions [4]–[6]. Expression changes of some of these genes have been used as sensitive indicators of the impact on the brain of situations leading to altered metabolism and/or transport of thyroid hormones, such as inactivation of the deiodinase genes *Dio2* and *Dio3*, thyroid hormone transporter genes *Mct8* and *Oatp1c1*, and the receptor genes *Thra* and *Thrb*, in addition to hypo or hyperthyroidism [5]–[9]. These studies have produced data that in some cases are difficult to explain, and reflect the complexity of the physiological mechanisms regulating gene expression *in vivo*. For example, inactivation of the *Mct8* gene which leads to restricted entry of T3 through the blood-brain barrier has a limited effect on cerebral cortex gene expression postnatally. This is due to up regulation of *Dio2* and increased local T3 formation. Consequently, compound inactivation of *Mct8* and *Dio2* leads to a situation more similar to hypothyroidism, but surprisingly some genes profoundly affected in the hypothyroid brain such as *Aldh1a1* are not affected by concomitant *Mct8* and *Dio2* inactivation [5]. Single inactivation of the *Dio2* gene in mice leads to brain T3 concentrations similar to hypothyroidism, but relatively minor effects on gene expression [10]. More recently it was found that *Dio2* inactivation preferentially affects expression of the genes regulated negatively by T3 rather than the positively regulated genes. The latter observation suggests that gene expression is also dependent on the route of entry of T3 to the brain [5]. Paradoxical effects of *Dio3* inactivation and T3 administration on cortex gene expression were also found [9]. Positive gene such as *Hr*, *Kcnj10*, *Shh*, and others were not affected by *Dio3* inactivation but were sensitive to T3-induced hyperthyroidism. In contrast negative genes such as *Cirbp*, *Fxyd6*, *Gpc3*, and others were sensitive to *Dio3* inactivation but resistant to T3 administration. These results could not be explained by a simple mechanism of neuronal T3 increase by decreased degradation in the *Dio3KO* mice, and indicate that the source of T3 is also important.

On the other hand, single or compound inactivation of the mouse *Thra* and *Thrb* genes leads to mild perturbations of gene expression compared to hypothyroidism. In a recent study [6] we found that from 27 cortex and striatal genes which expression was reduced by hypothyroidism only around 10 were reduced by TRα1 deficiency and 6 were increased by TRβ deficiency. Inactivation of both receptor genes increased the number of affected genes to 17, and there was no correlation between the effects of hypothyroidism and the effects of receptor gene inactivation. In general the conclusion of these studies was that in the absence of TRα1 thyroid hormone-dependent gene expression can be largely maintained by TRβ, and that the effects of hypothyroidism are largely due to the transcriptional effects of unliganded TR.

It is likely that thyroid hormone action on the brain *in vivo* is modulated by many interacting physiological factors acting on micro domains. The bulk effect observed *in vivo* in individual brain regions would be an aggregate result of the interaction of T3 with additional factors that may be modulated differently in each micro domain, depending on the cellular composition, expression of receptors, transporters, deiodinases, and the crosstalk with other signaling pathways. To better understand the role of different factors in the action of thyroid hormone on neural gene expression we have analyzed the expression of some selected genes in primary cultures of mouse cerebrocortical cells. Specifically we studied the effects of TR knockdown *in vivo* on the effects of T3 to better define the relative roles of TRα1 and TRβ. In addition we studied the crosstalk between thyroid hormone and retinoic acid, or glucocorticoids, as it is known that the effects of these hormonal systems are frequently interrelated. For example, retinoic acid and thyroid hormone may regulate the expression of some genes through similar responsive elements [11]–[13]. Examples of genes regulated by T3 and glucocorticoids at the transcriptional level include GH [14]–[17], cholesterol 7α-hydrolase [18], liver cytosolic PEPCK [19], FAS [20], *Pdk4* [21], and *Klf9* [22], among other genes.

## Materials and Methods

Ethics statement: All experimental procedures involving animals were performed following the European Union Council guidelines (directive 2010/63/UE) and Spanish regulations (R.D.1201/2005, and Law 32/2007). They were approved by the ethics committee of our institution (Consejo Superior de Investigaciones Científicas, CSIC; approval number SAF2011–25608).

Animals were housed in temperature (22±2°C) and light (12∶12 light-dark cycle; lights on at 7 a.m.) controlled conditions and had free access to food and water. Experiments involving comparisons between genotypes were from the strains of mice described elsewhere [6]. For the effects of dexamethasone (DEX) and retinoic acid (RA) we used cultures derived from mice of the C57BL/6J strain.

Pregnant dams were euthanized with CO2 on gestation day 17.5, and the fetuses were extracted and euthanized by decapitation. The cerebral cortices were dissected at in PBS containing 1% BSA and 0.1% Glucose. The tissue was disaggregated by enzymatic digestion with 0.4 mg/ml Papain (Roche) and passing through a 0.9-mm syringe in the presence of DNAse I. The homogenate was centrifuged and the cells were resuspended in serum free culture medium (Neurobasal Medium supplemented with 2% B27, Glutamax, 10 U/ml Penicillin, and 10 U/ml Streptomycin). The cells were seeded on poly-L-ornithine-coated 12-well multiwells (Sigma). The wells were previously preincubated with a mixture of Horse Serum and culture medium (1∶1), and Laminin (1 µg/ml) (Sigma). Cells were added to this media in culture medium (6×10^5^ cells per well). After 9 days, the cells were incubated 24 h in the same medium without B27 supplement before adding the different treatments: 1 nM T3, 1 µM RA, or 10 nM DEX. Incubation times are indicated in the figure legends. Control cultures without treatment were incubated in parallel. To examine the response to T3 in the presence of inhibition of protein synthesis, cycloheximide (CHX, Sigma) was added to the cultures at a concentration of 8 µg/ml 30 min before T3 (10 nM) and the cells were harvested 6 hours after T3 addition.

The composition of the primary cultures was analyzed by immunofluorescence as follows: Cells plated on glass coverslips were fixed with absolute acetone for 7 min at −20°C. After permeabilization for 5 min in 0.5% Triton X-100 in PBS, the cells were incubated for 1 h in 1% BSA 2% cold fish skin gelatin (Sigma) 0.05% Triton X-100 in PBS to block non-specific antibody binding sites. For immunofluorescence labeling, the cells were stained by overnight incubation at 4 C in the blocking buffer with the following combination of primary antibodies diluted1∶500: monoclonal anti glial fibrillary acidic protein (Clone G-A-5, Sigma) for astrocytes and rabbit polyclonal anti-NeuN [23] (Millipore) for neurons. The secondary antibodies were donkey anti mouse Alexa 488 (green) and donkey anti rabbit Alexa 555 (red) and were used at 1∶500 dilution. Cells were then washed in PBS and incubated for 10 min with 4′,6-diamidino-2-phenylindole (DAPI, Gibco® Life Technologies) 0.1 µg/ml in PBS to label the nucleus. Omitting the first antibodies in the incubation reaction gave no signal. Confocal images were acquired using an inverted Zeiss LSM 710 laser scanning microscope with a plan-apochromatic objective 63×/N.A 1.3. Sequential scanning mode was used to avoid crosstalk between channels. All images shown correspond to the maximum intensity projection of a z-stack. Images were processed with Zen 2009 software and Adobe Photoshop (Figure S1). To calculate the relative abundance of neurons and astrocytes in the different cultures antibody-labeled cells and DAPI-stained nuclei were counted in photographs taken using a 40× objective. A total number of 100–200 cells were counted in sextuplicate for each culture. The relative number of neurons and astrocytes were calculated as a percentage of DAPI-stained nuclei. The results are presented in Table S1. These cultures are enriched in neurons and there were no differences in the % cells stained as neurons in the three cultures. The % astrocytes were slightly higher in the TRα1^−/−^ and the TRβ^−/−^ than in the Wt, although only statistically significant when comparing Wt and TRα1^−/−^. As in other studies [24] less than 10% of the cells were not stained as astrocytes or neurons.

Total RNA was isolated using the Trizol procedure (Invitrogen, Carlsbad, CA). 250 ng RNA was used as template for the cDNA synthesis with the high-capacity cDNA reverse transcription kit (Applied Biosystems, Foster City, CA). PCR assays were performed using SYBR Green or TaqMan assays (Applied Biosystems) on a 7900 HT fast real-time PCR system. The PCR program consisted in a hot start of 95°C for 10 min, followed by 40 cycles of 15 s at 95°C and 1 min at 60°C. Expression of *RARβ*, *RXRα* and *Cyp26b1* was measured using SYBR Green quantitative PCR with the following primers: RARβ forward 5′GTGGGCATGTCCAAAGAGTC3′; RARβ reverse 5′GCTCCGCTGTCATCTCATAG3′; RXRα forward 5′CCGCTCCATAGCTGTGAAAG3′; RXRα reverse 5′CTGTTAGCACCCTGTCAAAG3′; Cyp26b1 forward 5′TACAGGGTTCCGGCTTCCAG3′; Cyp26b1 reverse 5′GCACCGGTCACACGAATCAG3. For analysis we used the 2-Ct method. For internal control RNAs we compared 18S RNA and Cyclophylin (*Ppia*) mRNA. Either control gave similar results (Figure S2). For the final calculations the data were corrected for 18S RNA and expressed relative to the value obtained for the control cells, which was given a value of 1.0.

To calculate significance of differences between means we used one- or two-way ANOVA, or two-tailed, unpaired Student’s *t*-test depending on the experiments. Calculations and graphics were performed using the GraphPad Prism software (www.graphpad.com/prism/).

## Results

The goal of this work was to analyze the effect of T3 on gene expression in cerebrocortical primary cultures. As T3 targets we selected genes previously known to be thyroid hormone dependent in brain, and that likely play a prominent role in mediating the effects of thyroid hormones on neural development, as follows: 1) the transcription factor and T3 receptor co-repressor *Hr* (Hairless), which was originally shown to be under transcriptional regulation by T3 in the cerebellum [25]. *Hr* has since then been a widely studied T3 target to analyze the effects of thyroid hormone on the brain. 2) The developmental morphogen *Shh* (Sonic hedgehog), which is regulated by thyroid hormone in the rat and mouse embryonic and adult brain, presumably at the transcriptional level [26]. 3)The transcription factor *Klf9* (Krüpfel-like transcription factor 9, also known as Basic Transcription Element Binding Protein or BTEB), which is regulated by thyroid hormone at the transcriptional level during tadpole metamorphosis, oligodendrocyte differentiation, and in the rodent brain [27]. 4) *Dio3*, which is regulated specifically by TRα1 [28]. 5) *Aldh1a1* (Aldehyde dehydrogenase 1a1), a gene sensitive to hypothyroidism produced by blocking thyroid hormone formation, but not by brain hypothyroidism resulting from *Mct8* and *Dio2* inactivation, as explained in the Introduction [5]. 6) *Aldh1a3*, a gene negatively regulated by T3 [29], which participates in the same retinoic acid synthesis pathway as *Aldh1a1* [30]. To search for clues that may explain the puzzling behaviors mentioned above we analyzed the thyroid hormone receptors subtypes specificity and also the effects of other factors such as retinoic acid, and glucocorticoids and their interactions with T3.

**Figure 1:**
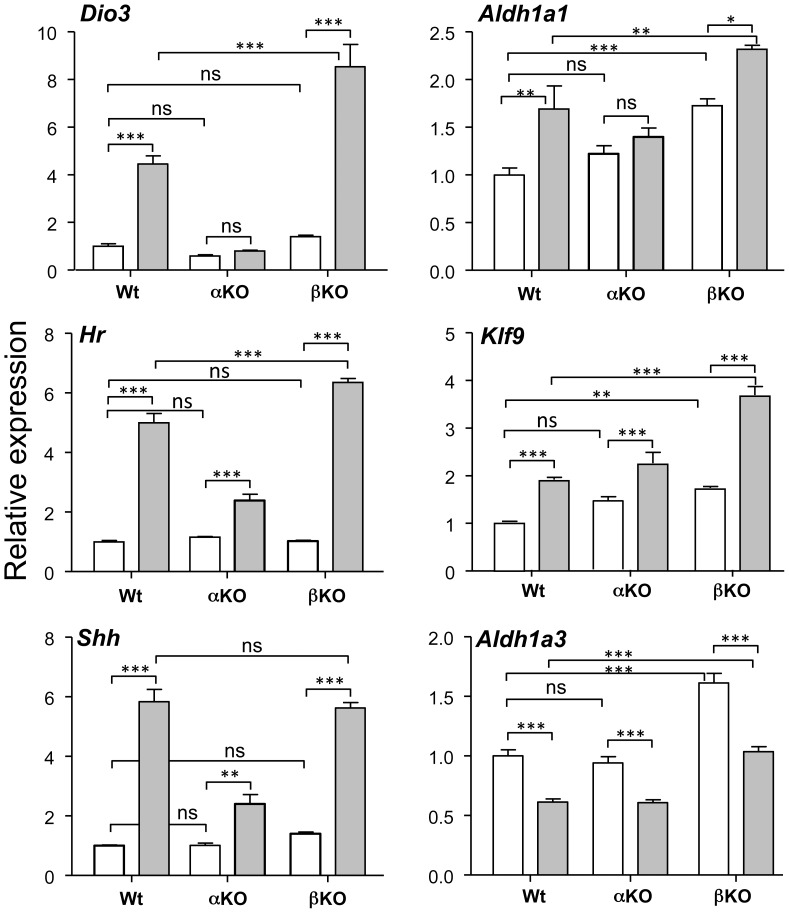
Effect of T3 on gene expression in primary mouse cerebrocortical cell cultures in the presence or absence of TRα1 or TRβ. Cells (n = 6) from wild type (Wt), TRα1 KO (αKO), or TRβ KO (βKO) mice were incubated for 24 hours in the absence (open bars) or in the presence of 1 nM T3 (filled bars). Statistical analysis was by two-way ANOVA; ns = P>0.05; * = P<0.05; ** = P<0.01; *** = P<0.001.

The relative role of receptor subtypes in the gene responses to T3 is illustrated in Figure 1. For this experiment we isolated E17.5 cerebrocortical cells from Wt mice, TRα1 KO mice, and TRβ KO mice. Gene expression was measured in the absence of T3, and 24 h after addition of 1 nM T3 to the cultures. In previous experiments (not shown) we found that increasing the T3 concentration up to 100 nM did not increase the response above that attained with 1 nM T3. As an experimental control of the effect of receptor inactivation we measured the expression of *Dio3* (encoding the type 3 deiodinase), which is regulated by T3 in a TRα1 isoform-specific way. *Dio3* expression in cells lacking TRα1 or TRβ was similar to Wt cells in the absence of T3. Addition of T3 increased *Dio3* expression by 4-fold in the Wt cells, and had no effect on the TRα1-deficient cells. TRβ deficiency potentiated the effect of T3, with an 8-fold induction, to twice the level obtained in the Wt cells. From the rest of the genes studied, only *Aldh1a1* was unresponsive in cells deficient of TRα1. T3 increased *Aldh1a1* expression in the Wt cells, but had no effect on the TRα1-deficient cells. In the TRβ-deficient cells the basal expression of *Aldh1a1* in the absence of T3 was increased with respect to the untreated Wt cells, and was further increased by T3.

*Hr*, *Klf9*, and *Shh* showed no TR isoform specificity, and were induced by T3 in the three culture types. *Hr* and *Shh* were induced by 5-fold and 6-fold, respectively in Wt cells and about 2-fold in the TRα1-deficient cells. Similarly *Klf9* increased 2-fold after T3 addition to the Wt cells and 1.3-fold in the TRα1-deficient cells. In the TRβ-deficient cells T3 induced a similar effect as in the Wt cells on *Shh*, but the effects on *Hr* and *Klf9* were increased.

The Aldh1a1 enzyme is involved in RA synthesis, catalyzing conversion of all-trans-retinal to all-trans-retinoic acid. Another aldehyde dehydrogenase Aldh1a3 is also involved in this pathway. In contrast to *Aldh1a1*, *Aldh1a3* is negatively regulated by T3 (Figure 1), with a 40% reduction after T3 addition to the Wt cells. TRα1-deficient cells behave exactly as the Wt cells. In the absence of TRβ the basal expression increased by about 60% and T3 induced a similar effect as in the Wt cells.

**Figure 2:**
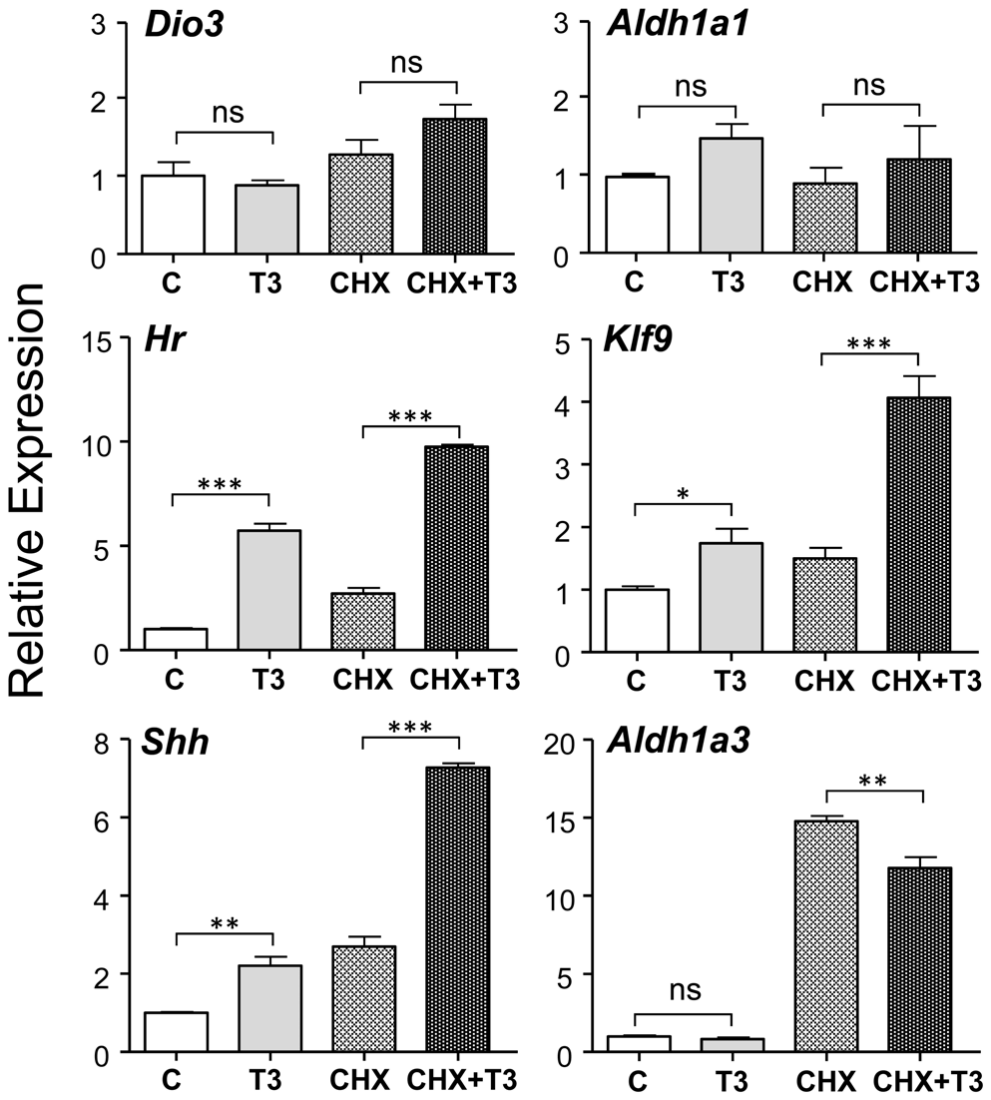
Effect of T3 in the presence or absence of cycloheximide (CHX) on gene expression in primary mouse cerebrocortical cell cultures from wild type mice. The cells (n = 4) were incubated for 30 min with or without CHX (8 µg/ml) before adding T3 (10 nM), and then incubated for 6 hours. Statistical analysis was by one-way ANOVA; ns = P>0.05; * = P<0.05; ** = P<0.01; *** = P<0.001.

To check whether the effects of T3 were due to a direct action on gene transcription, or mediated through increased expression of T3-dependent auxiliary proteins or transcription factors, we analyzed the effect of T3 in a shorter time, in the presence of the protein synthesis inhibitor CHX (Figure 2). After 6 hours in the presence of T3 an increased expression of *Hr*, *Klf9*, and *Shh* was already observed. The effect on any of these genes was not blocked by CHX pretreatment, indicating a direct effect of T3 on these genes. In contrast T3 had no significant effects on *Dio3*, *Aldh1a1* and *Aldh1a3* at 6 hours. Therefore no conclusions about the mechanism of induction could be made for *Dio3* and *Aldh1a1*. CHX had a strong stabilizing effect on *Aldh1a3* mRNA, and in the cells pretreated with CHX T3 was able to reduce *Aldh1a3* expression, suggesting that also for this gene the effect was direct.

**Figure 3:**
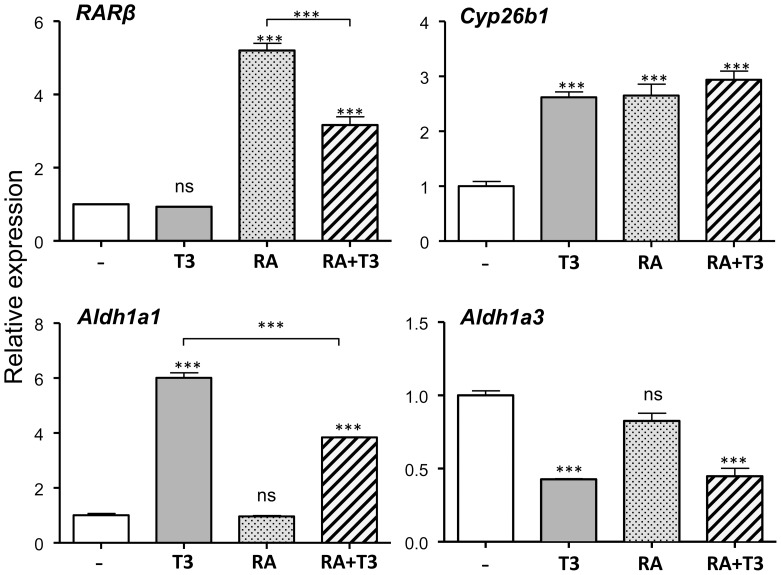
Effects of T3 and all-trans retinoic acid (RA) on gene expression in primary mouse cerebrocortical cell cultures from wild type mice. The cells (n = 4) were incubated for 48 hours in the absence or presence of T3 (1 nM), RA (1 µM) or both. Statistical analysis was by one-way ANOVA; ns = P>0.05; *** = P<0.001.

Since Aldh1a1 and Aldh1a3 are involved in RA synthesis we analyzed the effect of RA and a possible synergism with T3. As controls for the effects of RA we measured the expression of two RA-target genes, the RA receptor β, *RARβ* and the RA degrading enzyme *Cyp26b1* (Figure 3) [31]. *RARβ* was increased by RA but not by T3. When added together the presence of T3 inhibited the effect of RA. Interestingly we found that *Cyp26b1* was positively regulated by T3, with an effect quantitatively similar to that obtained with RA. There was no additive effect when both agents were added together. The relative effects of RA and T3 on *Aldh1a1* were a mirror image of those on *RARβ*. *Aldh1a1* was increased by T3 but without effect of RA, but RA inhibited the effect of T3 when added together. *Aldh1a3* was down regulated by T3 and there was no effect of RA.

**Figure 4:**
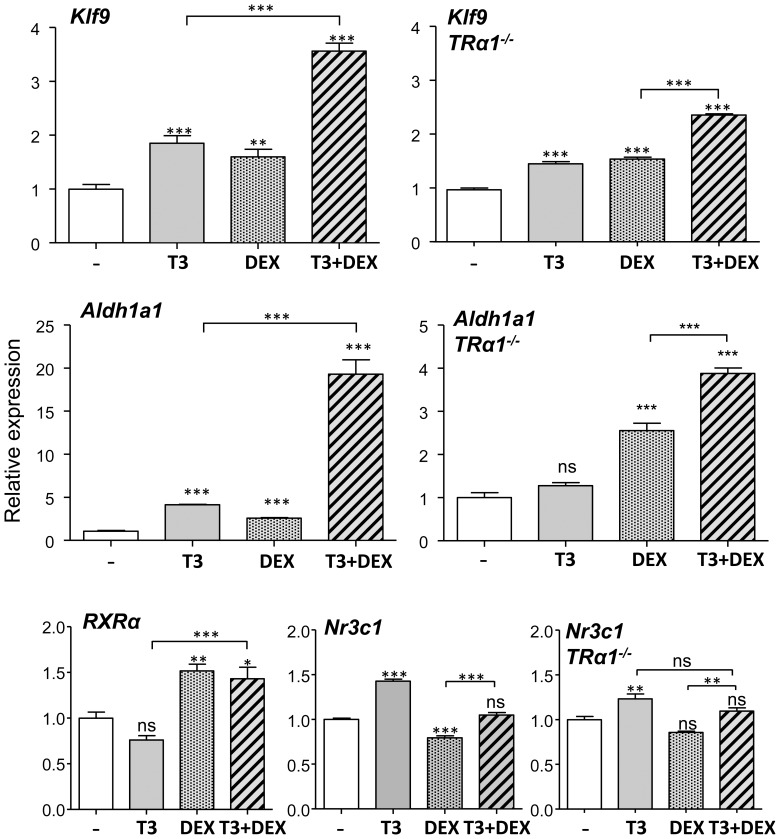
Effects of T3 and dexamethasone (DEX) on gene expression in primary mouse cerebrocortical cell cultures from wild type mice or from TRα1 KO mice. The cells (n = 4) were incubated for 48 hours in the absence or presence of T3 (1 nM), DEX (10 nM) or both. Statistical analysis was by one-way ANOVA; ns = P>0.05; * = P<0.05; ** = P<0.01; *** = P<0.001.

Many thyroid hormone regulated genes are also influenced by glucocorticoids, and in some instances there is synergism between the two hormones. Among the genes analyzed in this work, it is known that *Klf9* in tadpoles is synergistically regulated by T3 and glucocorticoids at the transcriptional level. Indeed we found that also on Wt cerebrocortical cells (Figure 4) DEX stimulated *Klf9* expression, similarly to T3, and there was a synergistic effect when both hormones were added together. *Aldh1a1* expression was also stimulated by dexamethasone, in addition to T3. In the presence of both hormones there was a strong synergistic effect of T3 and DEX and *Aldh1a1* expression was increased 20-fold. To explore possible mechanisms for the interaction between T3 and DEX we measured the effect of DEX on the TR heterodimeric partner RXRα, as it has been reported that DEX increases the expression of RXRα [32]. Indeed we found that DEX increased *RXRα* expression (Figure 4), but not *RXRγ* (not shown). In TRα1-deficient cells T3 and DEX also stimulated *Klf9* expression, as was expected due to lack of receptor specificity. However and in agreement with the previous finding, TRα1 was needed for the effect of T3 on *Aldh1a1* expression, since the response to T3 in cells derived from TRα1 KO mice was suppressed, whereas the response to DEX was maintained. Although T3 alone had no effect in the absence of TRα1, it increased the response to dexamethasone.

Among the possible mechanisms by which T3 potentiates the effect of DEX in the absence of TRα1, is an increased expression of the glucocorticoid receptor. To test this hypothesis we measured the effect of T3 on the glucocorticoid receptor mRNA *Nr3c1* in Wt cells and in TRα1-deficient cells (Figure 4). In both types of cells T3 increased *Nr3c1* expression by 30–50%. DEX alone decreased *Nr3c1* expression by 25%, but not when added together with T3. The effect of T3 was not observed at shorter times of incubation, i.e., 6 hours, and we cannot conclude that it is a direct action of the hormone (not shown).

## Discussion

In this work we have analyzed the contribution of factors others than T3 itself, that might be involved in the regulation of gene expression and that may play diverse roles *in vivo*. This experimental set up allowed us to confirm that genes which expression in the mouse cerebral cortex [6] is strongly dependent on thyroid hormones are indeed cellular targets of T3. The altered expression previously observed *in vivo* after induction of hypothyroidism was not due to a distal effect of the hypothyroid condition, but reflected a cellular effect of the hormone. The cellular system employed in this work is based on the culture of primary cells isolated from the embryonic mouse brain. These cultures are enriched in neurons but also contain astrocytes and unidentified cells (Table S1, Figure S1 and [24]). Therefore any effect observed by hormonal manipulations of these cells could have its origin in neurons or in astrocytes, depending among other factors on the relative expression of the target gene in each of these cell types.

First we analyzed the specific contribution of the TRα1 and TRβ receptor subtypes by measuring the response to T3 of primary cells derived from the cerebral cortex of TRα1 and TRβ KO mice. The results confirm [6] that at the cellular level both TRα1 and TRβ mediate the effects of T3, with two exceptions, *Dio3* and *Aldh1a1*. *Dio3* was already shown to be regulated specifically by TRα1 [28], and we confirm this fact in the cultured neurons. Also *Aldh1a1* appears to be regulated specifically by TRα1, since no induction by T3 was observed in cells derived from TRα1 KO mice. *Klf9* and *Aldh1a3* show no preference for each of the TR subtypes. A recent global analysis of TR specificity in HeLa cells expressing exogenous TRα1 or TRβ1 [33] concluded that there are no complete TR subtype specificity, although TRα1 or TRβ1 showed some gene preferences, depending on the time of exposure and the dose of T3. In established neural cell lines expression TRα1 or TRβ1 lead to substantial differences in the gene network regulated by T3, without correlation with differential chromatin occupancy [34].

Direct transcriptional regulation by T3, has previously been shown for *Hr* [25], *Klf9* [27], [35] *Dio3* [28], and *Shh* [26]. Chromatin occupancy by the TR has been shown for *Hr* and *Klf9* in established neural cell lines [34]. Here we confirm that also in cerebrocortical primary cells *Hr*, *Klf9*, and *Shh* are direct transcriptional targets of T3, given that the effect of the hormone was not blocked by previous treatment with CHX to inhibit protein synthesis. We could not determine whether *Dio3* was also transcriptionally regulated by T3 because it was not stimulated by T3 at 6 hours of incubation, preventing to test the effect of CHX. However, a specific TRα1 binding site has been demonstrated in the upstream region of the gene [28]. The fact that we could not determine whether the effect of T3 was direct, suggest the possibility that the full effect of T3 requires interaction with other intermediate proteins. *Dio3* is a transcriptional target of *Shh* [36] which, as mentioned above is a direct T3 target. This raises the possibility that rapid and full induction of *Dio3* by T3 is the result of a direct transcriptional effect of TRα1 potentiated by the T3-dependent accumulation of Shh protein.

A relevant new finding is the influence of T3 on the expression of genes involved in the synthesis and degradation of RA. We had previously observed a reduction of *Aldh1a1* expression in the brain of hypothyroid mice. By stimulating *Aldh1a1* expression T3 would increase RA synthesis. However, this effect would be counteracted by down regulation of *Aldh1a3* acting in the same pathway, and by up regulation of *Cyp26b1,* a gene encoding a RA degrading enzyme. During development *Aldh1a3* expression is maximal at E14, and decreases to very low levels near birth, whereas *Aldh1a1* rises postnatally in the telencephalon [37]. It is likely that these changes of expression are influenced by the thyroid hormone concentrations and TR expression changes occurring during development. In addition to this bulk effect, the net effect of thyroid hormone on RA production should depend on the relative expression of each enzyme in specific brain regions. Since the effect of T3 on *Aldh1a1* expression is TRα1 dependent, the net effect of T3 in specific cells would depend on the relative concentrations of TRα1 and TRβ. Aldh1a1 is involved in the regulation of the total number of dopaminergic neurons [38] and in the metabolism of dopamine and norepinephrine [39]. *Aldh1a3* is expressed in the ganglionic eminence where RA and thyroid hormone influence tangential neuronal migrations leading to the generation of cerebral cortex interneurons [40], [41]. These effects are likely to be important in the regulation of developmental events by T3.

The effect of T3 on *Aldh1a1* expression is also sensitive to RA. Although at the doses used RA in isolation does not influence *Aldhla1* expression, it inhibits the effect of T3. On the contrary, the RARβ response to RA is reduced by the presence of T3. *Cyp26b1*, which is sensitive to RA and T3, does not show an additive effect in the presence of both compounds, which might also be due to mutual interference. In this respect it is likely that the combined responses to RA and T3 are modulated by COUP-TF1, which is widely expressed in the nucleus of primary neurons (unpublished results from our laboratory). In neurons derived from embryonic stem cells COUP-TF1 represses T3 induction of the T3 target gene *Camk4* and confers RA sensitivity. This effect is mediated through a regulatory element containing overlapping binding sites for TR, RAR, and COUP-TF1 [13].

In our primary cultures *Klf9* and *Aldh1a1* expression were increased by DEX which also potentiated the effects of T3. *Klf9* is induced by corticosterone in the tadpole brain and in the *Xenopus laevis* fibroblast cell line XTC-2, and is proposed to mediate effects of glucocorticoids on neuronal structure and function [22]. To the best of our knowledge, *Aldh1a1* was not known to be induced by glucocorticoids. DEX had an effect comparable to that of T3. But when added together there was a synergistic effect and *Aldh1a1* expression increased by 20-fold, the largest increment effected by T3 that we have knowledge on any gene in neural cells.

**Figure 5:**
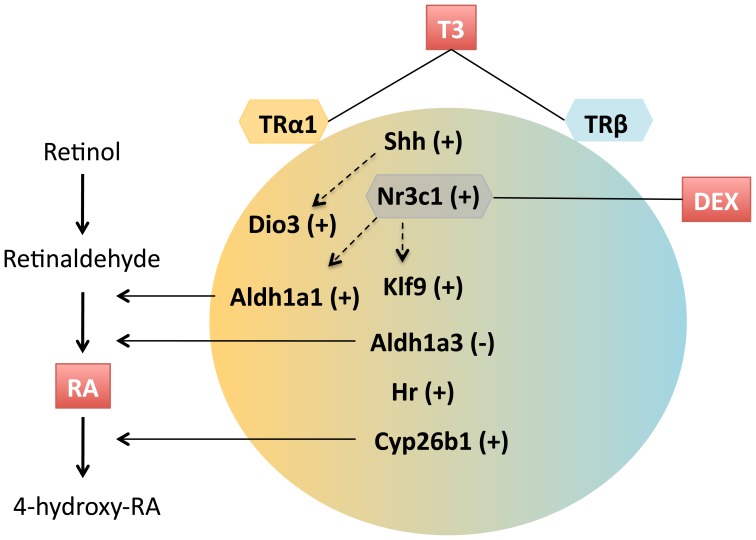
A summary of the interactions between T3 receptor subtype, and retinoic acid and glucocorticoid signaling in the T3-mediated control of gene expression described in this work. T3 induces up regulation (+) or down regulation (−) of gene expression. Specificity of TR subtypes is depicted by a color gradient. *Dio3* and *Aldh1a1* require TRα1, but no clear preferences were detected for other genes. Transcriptionally direct T3 responses include *Shh*, *Hr*, and *Klf9*. Induction of *Shh* is probably involved in *Dio3* regulation by T3. T3 regulates the expression of enzymes involved in the synthesis and degradation of retinoic acid (RA). The effect of T3 on *Klf9* and on *Aldh1a1* is potentiated by glucocorticoids, in part by up regulation of the glucocorticoid receptor *Nr3c1*.

A summary of the crosstalk between T3, RA and glucocorticoid signaling, as described in the present work is illustrated in Figure 5. These interactions are likely to be operating in different cell population micro domains at different stages of development. The net result would be a function of the expression of the different components present in the figure as the cells undergo their differentiation program.

## Supporting Information

Figure S1 **Confocal images of the cerebrocortical cultures from Wt, TRα1KO and TRβKo mice.** Upper panels: Cells stained with antibodies against GFAP and NeuN. Lower panels: Nuclei stained with DAPI. Scale bar = 25 µm.
(TIF)

Figure S2 **Correlation between qPCR measurements using as internal control 18S RNA or Cyclophylin RNA (** ***Ppia*** **).** Correlation coefficient was 0.95, P<0.0001.
(TIF)

Table S1 Cellular composition of the cerebrocortical primary cultures. Shown are the percentage of neurons and astrocytes relative to the total number of DAPI-stained nuclei. Data are mean ± SD (n = 6), and 95% Confidence Interval (CI). (*): P<0.05 compared to Wt. Other comparisons were not significant.
(DOCX)
