# Supplementary figures and images for: Thyroid Hormone Regulation of Gene Expression in Primary Cerebrocortical Cells: Role of Thyroid Hormone Receptor Subtypes and Interactions with Retinoic Acid and Glucocorticoids
Source: PLoS One. 2014 Mar 11;9(3):e91692. doi: 10.1371/journal.pone.0091692 (PMC3950245; doi:10.1371/journal.pone.0091692)

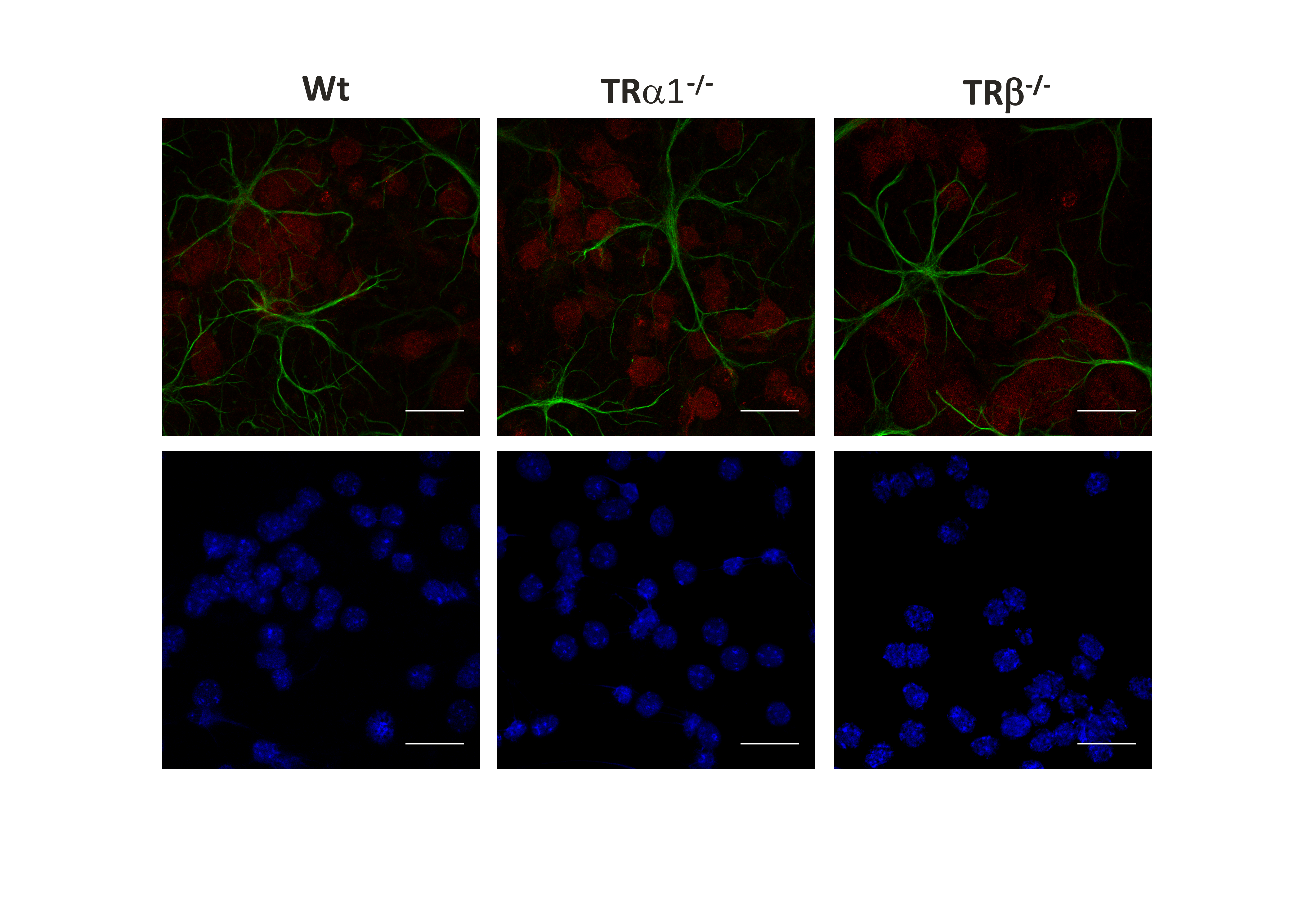

Supplement: Figure S1 — Confocal images of the cerebrocortical cultures from Wt, TRα1KO and TRβKo mice. Upper panels: Cells stained with antibodies against GFAP and NeuN. Lower panels: Nuclei stained with DAPI. Scale bar = 25 µm. (TIF) [file pone.0091692.s001.tif]

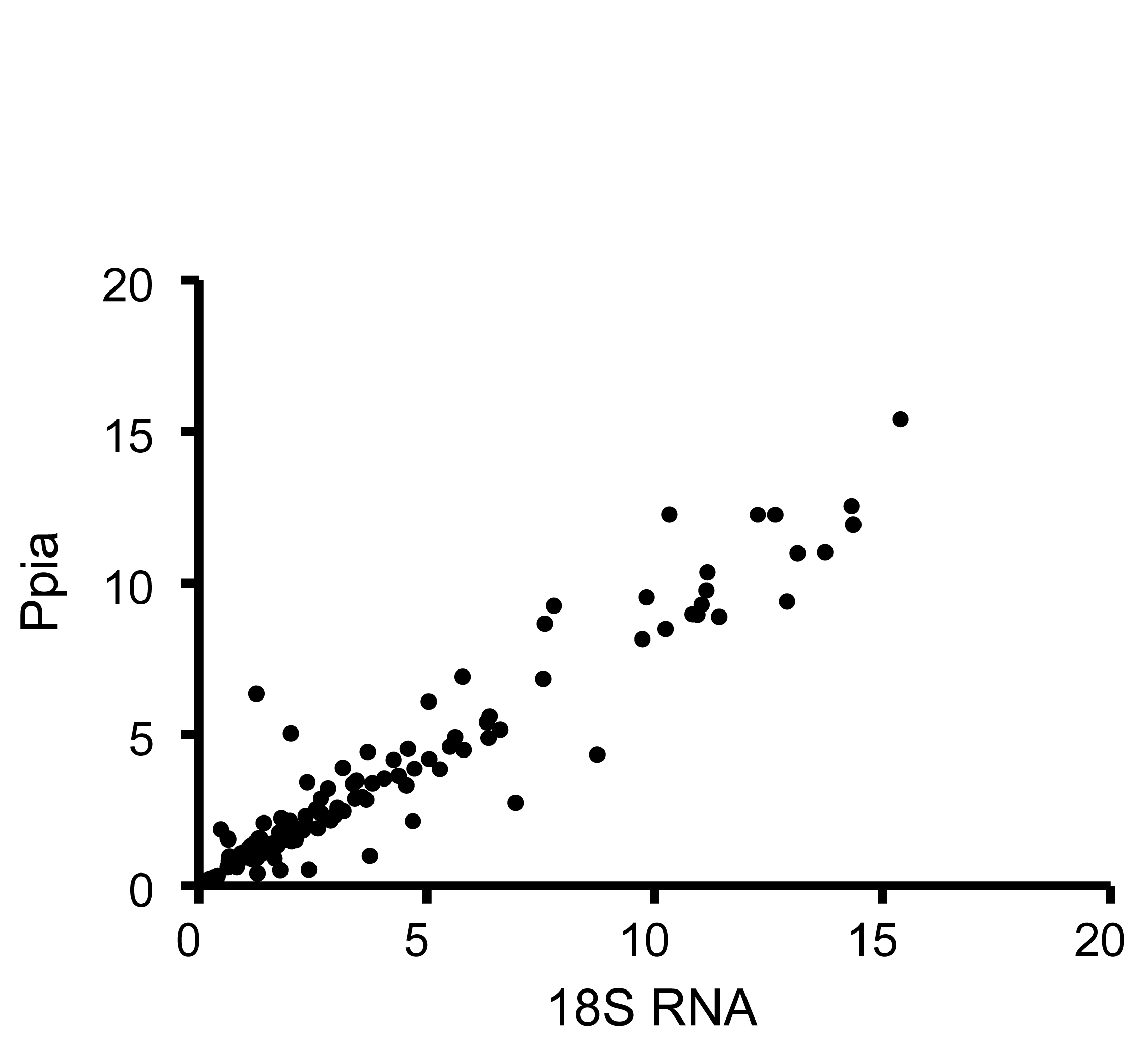

Supplement: Figure S2 — Correlation between qPCR measurements using as internal control 18S RNA or Cyclophylin RNA ( Ppia ). Correlation coefficient was 0.95, P<0.0001. (TIF) [file pone.0091692.s002.tif]
