# Supplementary material for: Thyroid Hormone Regulation of Gene Expression in Primary Cerebrocortical Cells: Role of Thyroid Hormone Receptor Subtypes and Interactions with Retinoic Acid and Glucocorticoids
Source: PLoS One. 2014 Mar 11;9(3):e91692. doi: 10.1371/journal.pone.0091692 (PMC3950245; doi:10.1371/journal.pone.0091692)
Supplement: Table S1 — Cellular composition of the cerebrocortical primary cultures. Shown are the percentage of neurons and astrocytes relative to the total number of DAPI-stained nuclei. Data are mean ± SD (n = 6), and 95% Confidence Interval (CI). (*): P<0.05 compared to Wt. Other comparisons were not significant. (DOCX) [file pone.0091692.s003.docx]

|  | % Neurons | 95% CI | % Astrocytes | 95% CI |
| --- | --- | --- | --- | --- |
| Wt | 74.3 ± 4.9 | 69.2 - 79.4 | 15.6 ± 2.2 | 14.5 – 33.2 |
| TRα1KO | 76.2 ± 3.6 | 72.4 – 80.0 | 19.3 ± 2.6 (*) | 16.5 - 22.0 |
| TRβ KO | 78.3 ± 4.3 | 73.8 – 82.9 | 18.8 ± 2.2 | 16.4 - 21.1 |
